# Thyroid B-Cell Lymphoma in the Background of Hashimoto's Thyroiditis: A Case Report and Literature Review

**DOI:** 10.7759/cureus.57359

**Published:** 2024-03-31

**Authors:** Abdullah M Ghafouri, Suzan Alzaidi, Bader B Al-Kaabi, Mohammed A Awadh, Dohaa Bakhsh, Abdullah Alharbi

**Affiliations:** 1 Department of Otolaryngology, Head and Neck Surgery, King Fahad Armed Forces Hospital, Jeddah, SAU; 2 College of Medicine, Umm Al-Qura University, Makkah, SAU; 3 College of Medicine, Faculty of Medicine, King Abdulaziz University, Jeddah, SAU; 4 Department of Pathology, King Fahad Armed Forces Hospital, Jeddah, SAU

**Keywords:** diffuse large b-cell lymphoma, primary thyroid lymphoma, lymphoma, otolaryngology, case report, thyroid

## Abstract

Primary thyroid lymphoma (PTL) is a rare type of thyroid cancer, comprising less than 5% of all thyroid cancer cases. PTL includes subtypes like diffuse large B-cell lymphoma (DLBCL) and mucosa-associated lymphoid tissue lymphoma (MALT). The connection between PTL and autoimmune diseases of the thyroid, particularly Hashimoto's thyroiditis, has gained recognition in recent years. Studies have indicated an increased incidence of PTL among individuals with Hashimoto's thyroiditis. However, effectively recognizing and managing PTL in the context of autoimmune thyroid diseases remains challenging. Further research and clinical experience are needed to develop comprehensive strategies for early detection and optimal management of this complex condition.

In a case involving an 88-year-old female diagnosed with diffuse large B-cell lymphoma, she presented with a complaint of persistent neck swelling for five years. The patient also experienced symptoms such as dysphagia, hoarseness of voice, obstructive sleep apnea, and choking attacks. Surgical resection of the neck swelling was successfully performed, and the patient was referred to the oncology department for further treatment.

Thyroid B-cell lymphoma is an exceedingly rare form of thyroid cancer, typically identified in individuals who have a history of Hashimoto's thyroiditis. The prognosis for thyroid B-cell lymphoma is generally unfavorable, and surgical intervention remains the primary treatment approach for such cases.

## Introduction

Thyroid cancer, encompassing various types, accounts for approximately 1% of all cancers. Among the four main types of thyroid cancer, papillary thyroid cancer is the most common, comprising about 85-90% of cases. Follicular thyroid cancer accounts for approximately 5-10%, while medullary thyroid cancer represents about 1-3% of cases. Anaplastic thyroid cancer is the rarest form, making up only about 1% of all cases [1,2].

Another less common type of thyroid cancer is thyroid diffuse large B-cell lymphoma (DLBCL), which belongs to the category of non-Hodgkin lymphomas affecting the thyroid gland. Although the overall prevalence of thyroid lymphoma is low, estimated at approximately 1% of all thyroid cancers, its occurrence has been noted to increase in recent years, particularly in cases where it emerges alongside Hashimoto's thyroiditis (HT) [3].

Previous research has explored the connection between Hashimoto's thyroiditis and thyroid lymphoma. A study published in the Journal of Current Opinion in Oncology examined the link between Hashimoto's thyroiditis and the risk of developing thyroid lymphoma. The study found that individuals with Hashimoto's thyroiditis had a significantly higher risk of developing thyroid lymphoma compared to those without the condition [4].

Given the strong association between thyroid lymphoma and Hashimoto's thyroiditis, this case report aims to present a detailed account of a patient who presented to King Fahad Armed Forces Hospital in Jeddah, Saudi Arabia, with thyroid DLBCL in the background of Hashimoto's thyroiditis, along with reviewing relevant literature on this topic. By reviewing previous studies, we hope to gain a deeper understanding of the prevalence of thyroid B-cell lymphoma, the potential risk factors associated with its development, and the importance of increased surveillance in patients diagnosed with Hashimoto's thyroiditis.

## Case presentation

We hereby describe a case of an 88-year-old female known for diabetes type 2, hypertension, osteoporosis, and hypothyroidism referred from primary health care to the otorhinolaryngology clinic complaining of neck swelling that started five years ago, first noticed by the family, increasing in size associated with dysphagia, hoarseness, obstructive sleep apnea, and choking attacks that increased in the past three months. The patient denied any history of night sweats, loss of appetite, and weight loss. Other systemic reviews were unremarkable. She had no history of neck radiation or a family history of cancer. No previous surgeries were done. The patient was seen by multiple home health care providers and was diagnosed with Hashimoto’s thyroiditis. 

On examining the patient, a large central neck swelling was seen, which was nontender and immobile. No palpable lymph nodes were appreciated. An indirect laryngoscopy was done that showed mobile vocal cords bilaterally with a patent airway. 

**Figure 1:**
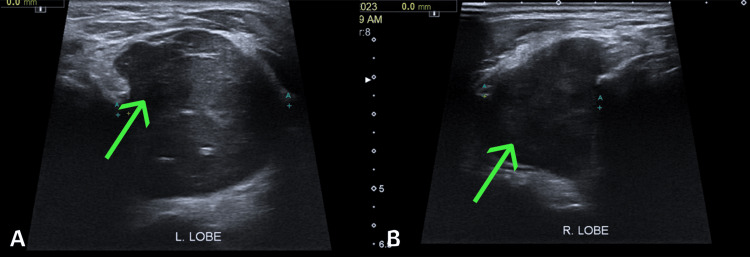
(A) Shows the largest nodule in the left thyroid lobe; (B) shows the largest nodule in the right thyroid lobe. (A) The arrow highlights the largest nodule in the left thyroid lobe, measuring 9.6 x 4.4 cm, TIRAD 4. (B) The arrow highlights the largest nodule in the right thyroid lobe, measuring 8.3 x 3.4 cm, TIRAD 4.

Ultrasound of the thyroid was reported as a multinodular thyroid with the largest nodule in the left lobe measuring 9.6 x 4.4 cm and in the right lobe measuring 8.3 x 3.4 cm (Figure 1). Bilateral TIRADs 4 were given to both nodules. Multiple bilateral benign-looking lymph nodes were appreciated. Fine-needle aspiration (FNA) was then done from both nodules. The right thyroid nodule was an unsatisfactory specimen, Bethesda 1. The left thyroid nodule was benign, Bethesda 2.

**Figure 2:**
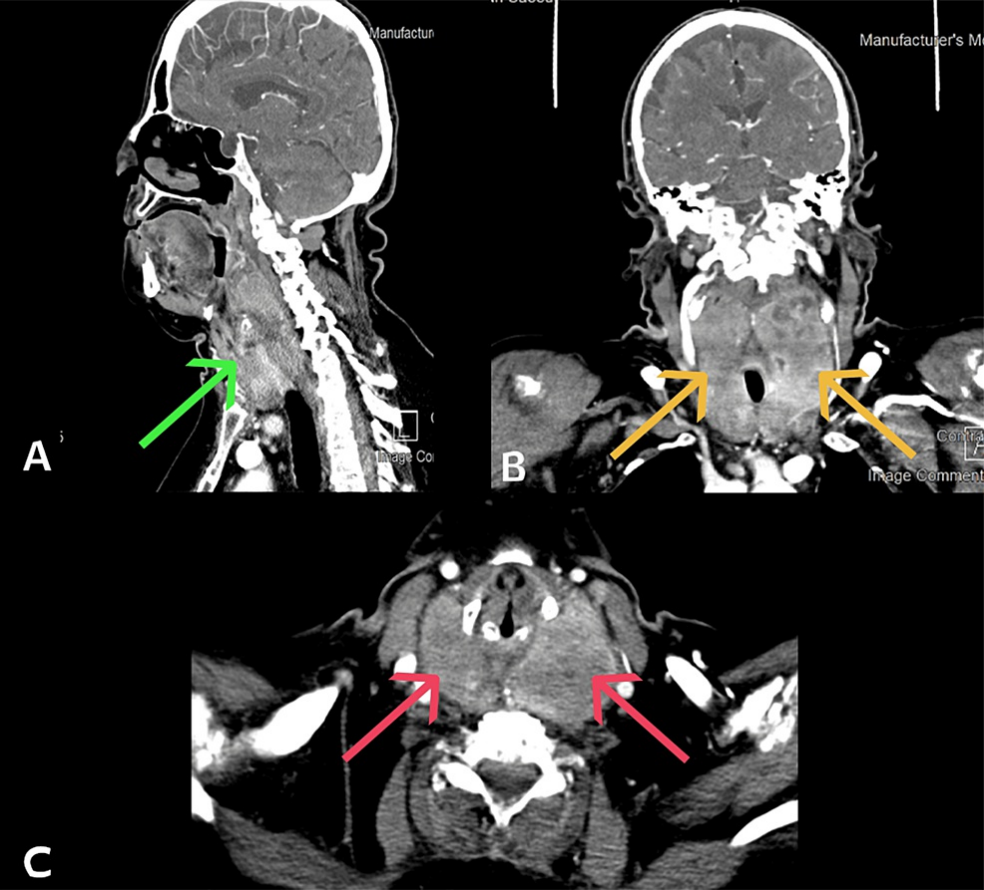
(A) A sagittal CT of the neck shows a thyroid mass; (B) an axial CT of the neck shows a bilateral thyroid mass; and (C) a coronal CT of the neck shows a bilateral thyroid mass. A: The green arrow shows a thyroid mass with no retrosternal extension; B: The orange arrow shows a bilateral thyroid mass with a significant mass effect on the trachea; and C: The red arrow shows a bilateral thyroid mass with a significant mass effect on the trachea.

Computed tomography scan (CT) was performed with no retrosternal extension. However, there appeared to be a significant mass effect on the trachea (Figure 2). 

**Figure 3:**
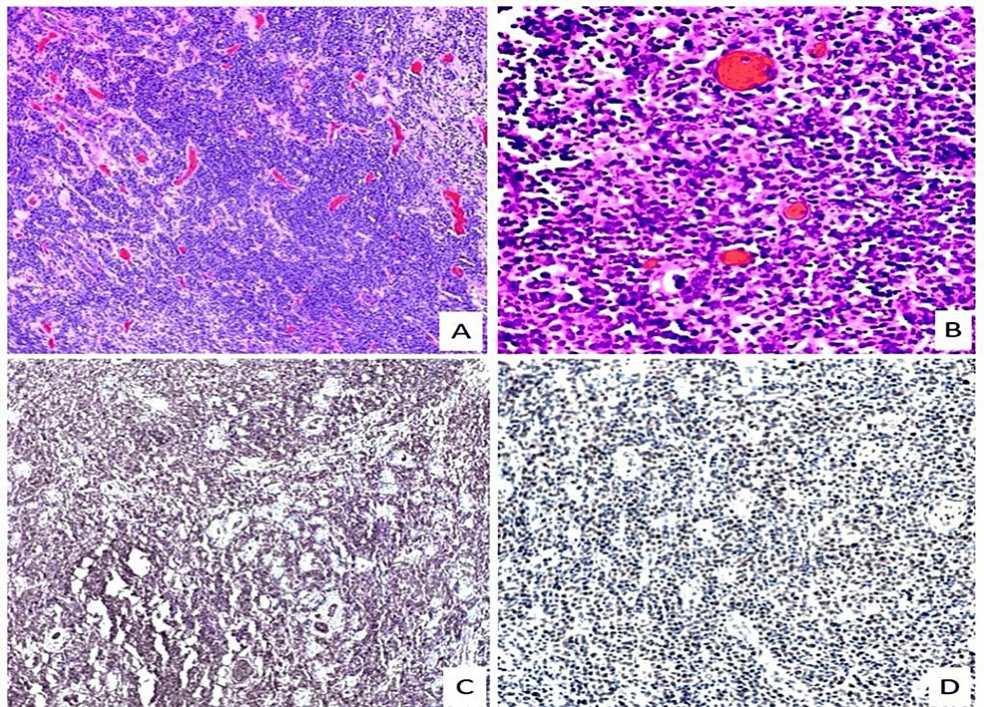
Histopathology of the thyroid specimens. A: Large atypical lymphocytes grow between nonneoplastic thyroid follicles (H&E stain at 100x). B: Diffuse infiltrate of large lymphocytes with prominent nucleoli and numerous apoptotic bodies (H&E stain at 400x). C: Lymphocyte infiltrate positive for CD79a marker on the immunohistochemical stain. D: Lymphocyte infiltrate positive for Bcl-6 marker on the immunohistochemical stain.

The patient then proceeded for total thyroidectomy due to the impact of the disease. The histologic examination of the surgical specimen showed the presence of non-Hodgkin diffuse B-cell lymphoma in the background of Hashimoto's thyroiditis (Figure 3). 

The patient was admitted to the intensive care unit (ICU) post-operatively for close monitoring for 24 hours. She had a normal voice and serum calcium levels throughout her stay in the hospital. The patient was discharged on her second day of stay in the hospital. One week afterward, the patient was seen in the clinic for follow-up and to break bad news. A referral to the oncology department was undergone so the patient could complete her treatment plan. 

## Discussion

Primary thyroid lymphoma (PTL) accounts for less than 5% of thyroid malignancies and less than 2% of extranodal lymphomas, making it a rare disease [5]. Non-Hodgkin lymphoma that originates from B-cells is classified into three categories, primarily DLBCL, mucus-associated lymphoid tissue lymphoma (MALT), or a mixed form (a combination of both), which accounts for the great majority of cases. The prognosis for MALT is more favorable than it is for DLBCL [6,7]. Lymphoblastic lymphoma and follicular lymphoma are fewer common types. T-cell lymphomas and Hodgkin lymphomas are also rare types of PTL [8]. The most common histological subtype of PTL, with a percentage ranging from 50 to 60%, is DLBCL [9]. PTL frequently affects middle-aged to older individuals, with a female-to-male ratio of 3:1 [3,4].

We found eight reported cases in the literature, with our current case being the 9th, and have analyzed common findings and outcomes within each paper (Table 1) [9-14]. The mean tumor size from available data was 6.13 cm, with the largest tumor measuring 7.5 cm [9]. It has a predilection in females with 71.4% of the cases that stated gender were women. The mean age of the cases was 63 years of age. PTL often happens in individuals with a history of Hashimoto's thyroiditis (HT), with 62.5% of the reported cases. One case of Down syndrome with HT was reported by Wei et al. Individuals with pre-existing HT are at risk between 40 and 80 times higher for developing primary thyroid lymphoma [15]. When a patient with a history of Hashimoto's thyroiditis presents with rapid and progressive enlargement of the neck mass, PTL should be suspected [5]. The most common presenting symptom was an enlarged neck mass of 50%. Shortness of breath, dysphonia, dysphagia, and an enlarged neck mass all have an equal percentage of 12.5%.

**Table 1:** Cases found in the literature. DWD: died with the disease; SOB: shortness of breath; MALT: mucosa-associated lymphoid tissue lymphoma.

Author, ref	Date	Gender	Age (years)	Presenting complaint	Tumor size in cm	Outcome	Treatment (surgery, chemotherapy, radiotherapy)	Other's conditions	Previous malignancy
Wei et al. [10]	2019	Female	43	Enlarge neck mass	8.1	DWD after three weeks	Surgery	Down syndrome, Hashimoto's thyroiditis	-
Chiang et al. [11]	2016	Female	61	Enlarge neck mass	5.5	Remission	Chemotherapy, radiotherapy	Hashimoto's thyroiditis	-
Akcali et al. [12]	2004	Male	50	Enlarge neck mass, SOB	6	Remission	Surgery	-	-
Marcy et al. [13]	2023	Female	91	Rapid progressive dysphonia, dysphagia	6.7	DWD after five months	Chemotherapy	Hashimoto's thyroiditis	Gastric MALT lymphoma
Liu et al. [14]	2011	Female	69	Enlarge neck mass	-	DWD after six months	Chemotherapy, radiotherapy	Hashimoto's thyroiditis	-
Liu et al. [14]	2011	Male	58	Enlarge neck mass	5.5	DWD after one year	Chemotherapy, surgery	Hashimoto's thyroiditis	-
Foppiani et al. [9]	2009	Female	70	Dysphonia	4.5	Remission	Surgery	Hashimoto's thyroiditis	-
Foppiani et al. [9]	2009	Female	65	Enlarge neck mass, dysphagia	7.5	Remission	Surgery	-	-

In diagnosing a patient with a rapidly enlarged neck mass, fine-needle aspiration (FNA) is the initial test; however, its limitations and insensitivity were noted [16]. The FNA is insufficient on its own [17]. Core-needle biopsy (CNB) is superior to FNA in the diagnosis of PTL with 94.3% than FNA with 61% to 42% [16]. However, with recent advances in immunophenotypic analysis, the accuracy of fine-needle aspiration (FNA) has improved in the diagnosis of PTL [18,19]. In diagnosing PTL, otolaryngologists may want to consider combining FNA and CNB. 50% of the cases were dead with the disease, with the longest duration of survival being 12 months after the diagnosis [14]. After undergoing the treatment plan, 50% of the cases experienced remission.

Our review showcases that 50% of cases underwent surgery as the primary treatment plan, combinations of chemotherapy and radiotherapy with 25%, chemotherapy alone with 12.5%, and chemotherapy and surgery with 12.5%. The main treatment modalities of PTL are multitherapeutic strategies with chemotherapy and radiotherapy, rituximab (IgG1), a chimeric monoclonal antibody against cell surface marker CD20 found on B-cells (R-CHOP), a recently added biological agent to the chemotherapy regimen cyclophosphamide, adriamycin (or doxorubicin), vincristine, and prednisone (CHOP), is used [20]. This is particularly relevant for the histologic subtype of DLBCL [17]. Dual-modality therapy has repeatedly demonstrated improved survival benefits over single-modality treatment [21].

## Conclusions

Thyroid B-cell lymphoma is an exceedingly rare form of thyroid cancer, typically identified in individuals who have a history of Hashimoto's thyroiditis. The prognosis for thyroid B-cell lymphoma is generally unfavorable, and surgical intervention remains the primary treatment approach for such cases.
